# Expression of Sirtuin 1 and 2 Is Associated with Poor Prognosis in Non-Small Cell Lung Cancer Patients

**DOI:** 10.1371/journal.pone.0124670

**Published:** 2015-04-27

**Authors:** Ivana Grbesa, María J. Pajares, Elena Martínez-Terroba, Jackeline Agorreta, Ana-Matea Mikecin, Marta Larráyoz, Miguel A. Idoate, Koraljka Gall-Troselj, Ruben Pio, Luis M. Montuenga

**Affiliations:** 1 Biomarkers Laboratory, Program of Solid Tumors and Biomarkers, Center for Applied Medical Research (CIMA), University of Navarra, 31008 Pamplona, Spain; 2 Group for Translational Medicine, Division of Molecular Medicine, Rudjer Boskovic Institute, 10000 Zagreb, Croatia; 3 Department of Histology and Pathology, School of Medicine, University of Navarra, 31008 Pamplona, Spain; 4 Navarra’s Health Research Institute (IDISNA), Pamplona, Spain; 5 Laboratory of Experimental Therapy, Division of Molecular Medicine, Rudjer Boskovic Institute, 10000 Zagreb, Croatia; 6 Department of Pathology, University Hospital and Faculty of Medicine, University of Navarra, 31008 Pamplona, Spain; 7 Laboratory of Epigenomics, Division of Molecular Medicine, Rudjer Boskovic Institute, 10000 Zagreb, Croatia; 8 Department of Biochemistry and Genetics, School of Science, University of Navarra, 31008 Pamplona, Spain; University of Arkansas for Medical Sciences; College of Pharmacy, UNITED STATES

## Abstract

**Background:**

Sirtuin 1 (SIRT1) and sirtuin 2 (SIRT2) are NAD+-dependent protein deacetylases involved in the regulation of key cancer-associated genes. In this study we evaluated the relevance of these deacetylases in lung cancer biology.

**Material and Methods:**

Protein levels of SIRT1 and SIRT2 were determined in non-small cell lung cancer (NSCLC) cell lines and primary tumors from 105 patients. Changes in proliferation were assessed after SIRT1 and SIRT2 downregulation in lung cancer cell lines using siRNA-mediated technology or tenovin-1, a SIRT1 and SIRT2 inhibitor.

**Results:**

High SIRT1 and SIRT2 protein levels were found in NSCLC cell lines compared with non-tumor lung epithelial cells. The expression of SIRT1 and SIRT2 proteins was also significantly higher in lung primary tumors than in normal tissue (P<0.001 for both sirtuins). Stronger nuclear SIRT1 staining was observed in adenocarcinomas than in squamous cell carcinomas (P=0.033). Interestingly, in NSCLC patients, high SIRT1 and SIRT2 expression levels were associated with shorter recurrence-free survival (P=0.04 and P=0.007, respectively). Moreover, the combination of high SIRT1 and SIRT2 expression was an independent prognostic factor for shorter recurrence-free survival (P=0.002) and overall survival (P=0.022). In vitro studies showed that SIRT1 and/or SIRT2 downregulation significantly decreased proliferation of NSCLC.

**Conclusions:**

Our results support the hypothesis that SIRT1 and SIRT2 have a protumorigenic role in lung cancer, promoting cell proliferation. Moreover, the expression of these proteins is associated with poor prognosis in NSCLC patients and may help to identify those NSCLC patients with high risk of recurrence that could benefit from adjuvant therapy after resection.

## Introduction

Lung cancer is one of the most common cancers worldwide and is the most frequent cause of cancer-related mortality [1]. Patients with localized disease are potentially curable by surgical resection, but 55–70% of these patients will relapse within 5 years of diagnosis. Adjuvant chemotherapy after complete resection is recommended in non-small cell lung cancer (NSCLC) stage II-IIIA patients to reduce the risk of recurrence and improve overall survival. However, the potential benefits of this therapy are contentious, especially in stage I patients, since there is not established criteria to discriminate patients that might benefit from those who might not or even could be harmed by adjuvant treatment. Molecular markers that accurately classify early NSCLC patients into high or low risk groups of developing post-resection recurrence will help to decide whether a specific lung cancer patient should receive or not adjuvant therapy after resection.

The mammalian sirtuin protein family comprises seven members which differ in subcellular localization and enzymatic activity. The best known members of this family are sirtuin 1 (SIRT1) and sirtuin 2 (SIRT2), two NAD-dependent deacetylases that are involved in many cellular processes including cell proliferation, cell death, senescence and stress response. In cancer, both sirtuins might play a promoting or suppressing role, depending on the organ or even the species [2]. In lung cancer, previously published results about the role of SIRT1 and or SIRT2 have not provided a clear and definite answer. SIRT1 downregulation induces cell growth inhibition, cell cycle arrest and/or apoptosis in NSCLC cells [3–6]. Also, nuclear SIRT1 expression has been associated with advanced tumor invasion, high pathological T stage and lymph node metastasis in NSCLC [7]. Interestingly, a recent study by Zhang et al. have showed that cytoplasmic SIRT1 expression predicts prognosis and response to chemotherapy in advanced NSCLC [5]. In contrast, SIRT1 activation has been reported to hamper lung cancer metastasis [8] and sensitize NSCLC cells to anticancer drugs [9]. Contradictory data have also been published on the role of SIRT2 in lung cancer. On one hand, SIRT2 overexpression inhibits cell growth and induces apoptosis in A549 and H1299 cells [10]. On the other hand, treatment with different SIRT2 inhibitors induces apoptosis [11] or enhances the chemosensitivity of NSCLC to etoposide treatment [12].

Therefore, the role of SIRT1 and SIRT2 in lung cancer remains unclear. To address this question we analyzed the prognostic value of the expression of these sirtuins (alone and in combination). We also explored the effect of SIRT1 and SIRT2 inhibition in cell proliferation, cell cycle arrest and apoptosis of NSCLC cell lines.

## Materials and Methods

### Patient samples

The initial study cohort consisted of 179 patients diagnosed with lung cancer who underwent surgical resection at Clínica Universidad de Navarra (Pamplona, Spain) between April 2000 and June 2010. Tissue specimens were examined and histologically classified using the 2004 WHO classification system for lung cancer [13]. Among these patients, we have included in this study 105 patients meeting the following criteria: NSCLC histology, absence of cancer within the five years prior surgery, no adjuvant therapy before surgical resection, enough tissue to perform the study and completeness of clinical data (resection data, histology, stage, smoking history and follow up data). A total of 60 patients were treated only with surgery and 45 with surgery followed by adjuvant treatment. The median follow up period was 45 months (interquartile range: 78 months–24.5 months). The characteristics of the study population are summarized in Table 1. The study protocol (IRB n° 005/2003) was approved by the institutional ethics committee of the University of Navarra. Written informed consent was obtained from each patient (as outlined in PLOS consent form) to publish these case details. Reported recommendations for tumor marker prognostic studies (REMARK) criteria were followed throughout the study [14].

**Table 1:** Clinicopathological features of 105 NSCLC patients.

Patient characteristics	*n* (%)
**Age (years)**	
median±SD	63±10
**Gender**	
Male	93 (88.6)
Female	12 (11.4)
**Histology**	
ADC	47 (44.8)
SCC	50 (47.6)
Other	8 (7.6)
**Stage**	
I	65 (61.9)
II	27(25.7)
III	9 (8.6)
IV	4 (3.8)
**Nodal (N) stage**	
N0	75 (71.4)
N1	23 (21.9)
N2	7 (6.7)
**Histological grade**	
WD	12 (12)
MD	45 (46)
PD	42 (42)
**Smoking history**	
Never	5 (4.8)
Former	74 (70.5)
Current	26 (24.7)
**Adjuvant therapy**	
Yes	45 (42.9)
No	60 (57.1)

ADC, adenocarcinomas; SCC, squamous cell carcinoma; WD, well differentiated; MD, moderately differentiated; PD, poorly differentiated; SD, standard deviation.

### Cell culture

Human NSCLC cell lines (A549, H1299, H157, H358, H460 and H520) were obtained from the American Type Culture Collection and the European Collection of Cell Cultures. All the cell lines were grown in RPMI complete medium: RPMI-1640 with L-glutamine (Lonza) supplemented with 10% Fetalclone III (Hyclone) and 1% Penicillin-Streptomycin (Lonza). Cells were authenticated by PCR analysis on the basis of their known mutations (COSMIC database).

Immortalized human bronchial epithelial cells (HBEC-3KT) were a kind gift from Dr. John D. Minna (University of Texas Southwestern Medical Center, Dallas, Texas). Cells were derived from a donor after obtaining informed consent on institutional review board-approved protocols. These non-tumorigenic cells were obtained from bronchial cells by overexpressing CDK4 and TERT [15,16]. They were grown in serum-free Keratinocyte-SFM (Gibco) supplemented with bovine pituitary extract (Gibco), recombinant EGF (Gibco) and 1% Penicillin-Streptomycin. Cells were cultured in a humidified incubator at 37°C and 5% CO_2_.

### Immunohistochemical analysis

Immunohistochemical assay to assess SIRT1 and SIRT2 protein expression was performed on formalin-fixed paraffin-embedded tissue sections. Endogenous peroxidase activity was quenched with 3% H_2_O_2_ for 10 min. Heat-mediated antigen retrieval was carried out in a Lab Vision PT module for 20 minutes either with EnVision FLEX target retrieval solution, high pH (Dako) for SIRT1 or citrate buffer (pH 6) for SIRT2. Sections were incubated overnight at 4°C with anti-SIRT1 (polyclonal antibody, Santa Cruz Biotechnology, sc-15404, 1/200) or anti-SIRT2 (monoclonal antibody, Santa Cruz Biotechnology, sc-28298, 1/50) antibodies. After applying the EnVision^+^ System-HRP (Dako) for 30 min, immunostaining was developed by incubation with Liquid DAB^+^ Substrate Chromogen System (Dako). Tissues expressing different levels of antigen were included in each immunohistochemical run to control for experimental variation. The slides were counterstained with Harris hematoxylin solution. Negative controls consisted in omission of the primary antibody or incubation with an isotype control antibody. The specificity of the SIRT1 and SIRT2 antibodies was demonstrated by Western blotting and immunocytochemistry of cell lines after SIRT1 or SIRT2 inhibition with specific siRNAs.

Staining scores were established by semiquantitative analysis [17]. Briefly, the extension and intensity of the staining were evaluated independently by two experienced observers (M.J.P. and E.M.) blinded to the clinical features and outcomes of patients. The extension of the staining was scored as the percentage of positive cells (0–100%). The intensity of staining was assessed by comparison with a known external positive control (0, below the level of detection; 1, weak; 2, moderate; 3, strong). A final score, called the H-score, was calculated by the summarization of the product of staining intensity by extension at each intensity level, as previously described [17]. Discordant independent readings were resolved by simultaneous review by both observers. Median values were used as the cut-off to distinguish between high expressors and low expressors. For the combined variable SIRT1&SIRT2, patients were divided in two groups: patients with high expression of both sirtuins and patients with low expression in at least one of the two sirtuins.

### Reverse transcription and real-time PCR

Total RNA was extracted according to the manufacturer's recommendations (RNeasy Kit, Qiagen). One μg of DNA was treated with DNase I (Invitrogen) and reverse transcribed using SuperScript III kit (Invitrogen). Real-time PCR was performed on Applied Biosystems 7300 Real-Time PCR System. Amplification mixture (20 μL) consisted of SYBR Green PCR Master Mix (Applied Biosystems) and 0.15 μM of each primer. Importin 8 (IPO8) mRNA expression was used as the endogenous control [18]. Primer sequences were: 5´CAGTGGCTGGAACAGTGAGA 3´ (SIRT1/F); 5´AGCGCCATGGAAAATGTAAC 3´ (SIRT1/R); 5´ ggaggaggcatggactttga 3´ (SIRT2/F); 5´ CATCCAAGGAGCTCAGCAAG 3´ (SIRT2/R); GACTCTCAGGGTCGAAAACGG (CDKN1A/F); GCGGATTAGGGCTTCCTCTT (CDKN1A/R); 5´GATTATGCTTCTCCCACCACA3´ (IPO8/F) and 5´AGGGCTCCATCTTTCTTCCT3´ (IPO8/R).

### Western blotting

Cells were lysed in RIPA buffer (10 mM Tris-Cl pH 7.4, 0.5 M NaCl, 1% sodium deoxycholate, 0.1% SDS, 1% Triton X-100) supplied with protease inhibitor cocktail (Complete Tablets, Roche). Protein concentration was determined by Pierce BCA Protein Assay Kit according to the manufacturer’s instructions (Thermo Scientific). Proteins were denatured in SDS sample buffer (Bio-Rad) at 95°C for 5 min, resolved by SDS-PAGE on NuPAGE Novex 4–12% Bis-Tris gels (Invitrogen) and transferred to nitrocellulose membranes (0.45 μm pore size, Bio-Rad). The membranes were blocked in 5% non-fat milk for an hour and incubated overnight at 4°C with the primary antibodies for SIRT1 (1/10,000), SIRT2 (1/500), TP53 (1/1,000) and β-actin (1/20,000). Secondary antibodies were applied (GE Healthcare, NA934; NA931) and chemiluminescent detection was performed using the Lumi-Light PLUS (Roche).

### Silencing with siRNAs

A549 and H1299 cells (80,000/well) were seeded in 6-well plates. The following day, the cells were transfected with siRNAs (1 nM) targeted against SIRT1 (SIRT1 siRNA #1 and #2) (Sigma-Aldrich) or SIRT2 (SIRT2 siRNAs #1 and #2) in Opti-MEM Reduced Serum Medium, GlutaMAX (Gibco) by Lipofectamine 2000 (Invitrogen). The siRNA sequences were siSIRT1# 1: 5’-GGAUAGGUCCAUAUACUUU[dT][dT]-3’; siSIRT1 # 2: 5’-CCACCUGAGUUGGAUGAUA[dT][dT]-3’; siSIRT2 #1: 5’-GCCAACCAUCUGUCACUACUU[dT][dT]-3’ and siSIRT2 #2: 5’-GCCCAAGTGTGAAGACTGTCA-3’. As a negative control, a scrambled siRNA (Dharmacon) was used. To minimize the off-target effects, preliminary experiments were performed to determine the minimal concentration of the siRNAs needed.

### Cellular proliferation assays

Cell viability was measured by thiazolyl blue tetrazolium bromide (MTT) assay. Cells were seeded in 96-well plates. When indicated they were treated with 10 μM Tenovin-1 (tnv-1, Cayman Chemical) or were transfected with siRNAs. After the specified period of time, MTT solution (0.5 mg/mL; Sigma-Aldrich) was added. The formazan crystals were dissolved in an extraction buffer (50% dimethylformamide and 20% SDS, pH 4.7). The absorbance (540/690 nm) was measured in a SunRise plate reader (Tecan).

For clonogenic assays, cells were seeded in 6-well plates at 300–400 cells/well, incubated overnight and treated with 10 μM tnv-1 or with its corresponding solvent (DMSO). After 10–14 days, the medium was removed. The cells were fixed in 3.7–4.0% formaldehyde (Panreac) and stained with 1.2% crystal violet (Sigma-Aldrich). The colonies were counted by using ImageJ software (NIH).

Anchorage independent cell growth was determined by soft agar colony formation assay. NSCLC cells were either treated with 10 μM tnv-1 for 72 h or the corresponding solvent control. Top layer solution (1 mL/well in a 6-well plate) consisted of 0.3% agar (in RPMI complete medium) and 1,000–10,000 cells/well. The bottom semisolid layer (2 mL/well of 6-well plate) consisted of 0.6% agar (in RPMI complete medium). After 10-day incubation, MTT solution (1.67 mg/mL) was added. The formed formazan crystals were dissolved in 0.5 mL of DMSO. The plates were scanned and the colonies were counted using ImageJ software (NIH).

### Cell cycle analysis

Propidium iodide (PI) staining followed by flow cytometric analysis was performed to determine the effects of tnv-1 on the cell cycle of the treated cells. NSCLC cells were plated at 50,000–100,000/well in a 6-well plate. The next day tnv-1 (10 μM) was added. After 72 h, cells were fixed overnight in cold 70% ethanol, incubated with 0.2 mg/mL RNase A (Sigma-Aldrich) for one hour at 37°C and stained with PI (10 μg/mL, Sigma-Aldrich). The PI fluorescence was collected from 20,000 events in FL3-H channel (linear scale) on FACSCalibur Flow Cytometer (Becton Dickinson). The obtained results were analyzed using ModFit software version 2.0 (Verity Software House) and FlowJo software v. 7.2.5 (Tree Star, Inc.).

### Annexin V assay

The cells were treated with 10 μM tnv-1 as described above. Forty-eight hours after the treatment, both floating and attached cells were collected and stained with Annexin V labeled with FITC (Beckton-Dickinson) and PI (Sigma-Aldrich) according to the manufacturer’s recommendations. The cells were analyzed in a FACSCalibur flow cytometer and the obtained results were analyzed using FlowJo software.

### Time-lapse confocal microscopy

H358 cells were seeded in an uncoated ibidi 8-well μ-slide chamber at 12,500 cells/well. Next day they were treated with tnv-1 (10 μM) or DMSO. The images were taken using an AxioCam MRm CCD camera (Carl Zeiss) mounted on to a Cell Observer confocal microscope (Carl Zeiss) under 10x magnification (N-Achroplan objective, Carl Zeiss). Photographs were taken every hour (four positions per well) for 72 h. Following acquisition, individual images were processed and converted into time-lapse movies with ImageJ software. The exact cell number per frame was automatically counted by using ImageJ plug-in programmed at our institutional imaging core facility.

### Statistical analysis

Student’s *t*-test (two-tailed, unpaired) was used for statistical analysis of the results of MTT assay, soft agar colony formation assay and time-lapse confocal microscopy after treatment with tnv-1.

The Spearman correlation coefficient testing was used to evaluate the association between SIRT1 and SIRT2 expression detected by immunohistochemistry. Wilcoxon’s signed-rank sum test was applied to analyze differences between normal and tumor tissues. The relationship between SIRT1 or SIRT2 protein expression (assayed by immunohistochemistry) and clinicopathological factors was studied by Pearson’s chi-squared (χ2) test. Patients were stratified into two groups according to the median values of the H-score. Cumulative survival of patients was estimated using Kaplan-Meier curves, and significant differences between groups were tested using the log-rank test. Recurrence-free survival (RFS) and overall survival (OS) were calculated from the date of surgery to the date of recurrence or death, respectively. Univariate and multivariate Cox proportional hazards analyses were used to assess the prognostic role of SIRT1 and SIRT2. Only those variables with *P* < 0.1 in the univariate analysis were included in the multivariate analysis. The proportional hazards assumption was examined by testing interactions between the covariables of the final model and time. A *P* value < 0.05 was considered as statistically significant (* and ** indicate *P* < 0.05 and *P* < 0.01, respectively). Statistical analysis was done using the SPSS software package (v. 15.0).

## Results

### Expression and significance of SIRT1 and SIRT2 in NSCLC cell lines and tumors

**Fig 1:**
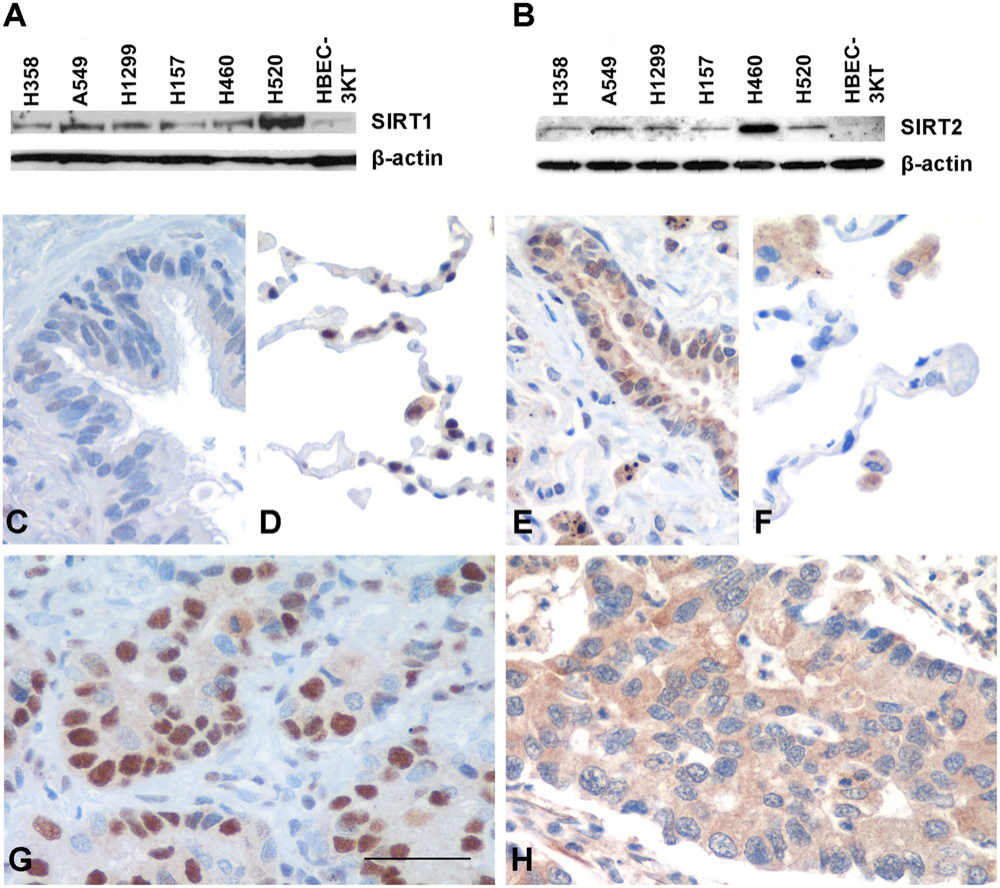
SIRT1 and SIRT2 are upregulated in NSCLC. (A, B) Immunoblotting of SIRT1 (A) and SIRT2 (B) in NSCLC cell lines and normal immortalized lung epithelial HBEC-3KT cells. The figure is representative of three different experiments. β-actin was used as control. (C-H) Representative immunohistochemical staining for SIRT1 (C, D, G) and SIRT2 (E, F, H) in normal human lung (C-F) and tumor (G, H) specimens expressing different levels of SIRT1 and SIRT2 proteins. In tumors, SIRT1 expression is predominantly nuclear whereas SIRT2 immunoreactivity is mostly localized in the cytoplasm. Bar = 50 μm.

We first evaluated the expression of SIRT1 and SIRT2 in a panel of human NSCLC cells and in HBEC-3KT by Western blotting and real-time PCR (Fig 1A, 1B and S1 Fig). SIRT1 and 2 protein levels were clearly higher in cancer cell lines than in immortalized epithelial cells (Fig 1A and 1B), although these differences were not observed at mRNA level (S1 Fig). To study the significance of SIRT1 and SIRT2 overexpression in human lung cancer, we analyzed their protein levels in a cohort of 105 NSCLC patients by immunohistochemistry. In normal tissues, SIRT1 and SIRT2 were expressed in the nuclei and the cytoplasm of some epithelial cells in bronchi and bronchioles (Fig 1C–1E) and alveolar macrophages (Fig 1D–1F). SIRT1 immunostaining was also present in pneumocytes (Fig 1D). In tumor cells, SIRT1 appeared predominantly in the nucleus of the cells (Fig 1G) whereas SIRT2 was located mainly in the cytoplasm (Fig 1H). The H-score analysis of the immunohistochemical signal showed a significant increase of SIRT1 and SIRT2 expression in tumors compared with their normal counterparts (*P*<0.001). We did not find association between the expression of SIRT1 and SIRT2 in tumor cells (*P* = 0.073, r = 0.170). The relationship between SIRT1 or SIRT2 expression and the clinicopathological characteristics of the patients was evaluated (S1 Table). No association was found except for SIRT1 and histology. SIRT1 expression levels were significantly higher in adenocarcinomas (ADC) than in squamous cell carcinomas (SCC) (*P* = 0.033; S1 Table).

**Fig 2:**
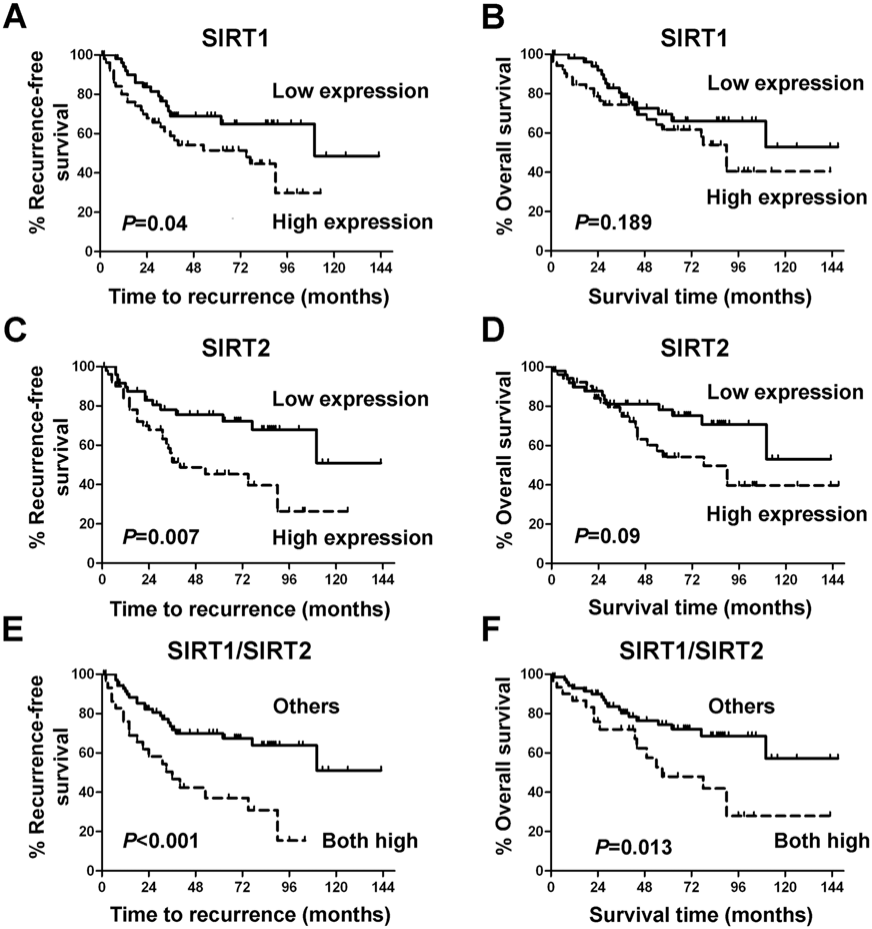
Combination of high levels of SIRT1 and SIRT2 proteins predicts shorter RFS and OS. Kaplan-Meier curves of RFS (A, C, E) and OS (B, D, F) for SIRT1 (A, B), SIRT2 (C, D) and the combination of SIRT1 and SIRT2 (E, F) as assessed by immunohistochemical staining.

Next, we analyzed whether sirtuin expression was associated with outcome in NSCLC patients. For SIRT1, patients with high levels of this protein showed significantly shorter RFS than patients with low levels (*P* = 0.04, Fig 2A). High SIRT2 expression was also associated with a significant decrease in RFS (*P* = 0.007, Fig 2C). No significant association between OS and SIRT1 or SIRT2 was found in our series (SIRT1: *P* = 0.189; SIRT2: *P* = 0.09; Fig 2B–2D). We also evaluated the prognostic capacity of the combination of SIRT1 and SIRT2 expression. Patients with high SIRT1 and SIRT2 showed significantly shorter RFS (*P*<0.001) and OS (*P* = 0.013) than patients with low levels of at least one sirtuin (Fig 2E and 2F). To investigate whether these striking results were dependent on tumor histology, ADC and SCC subgroups were analyzed separately. In spite of the reduced number of patients remaining in each subset, similar results were found in both histologies (S2 and S3 Figs).

As shown in Table 2, multivariate analysis, adjusted by stage, pointed out the combination of SIRT1 and SIRT2 as an independent prognostic factor for poor RFS (*P* = 0.002; HR = 2.698; 95%CI = 1.457–4.996) and OS (*P* = 0.022; HR = 2.193; 95%CI = 1.118–4.300) in NSCLC.

**Table 2:** Univariate and multivariate Cox proportional hazards model for recurrence-free survival (RFS) and overall survival (OS).

	n (%)	RFS				OS			
		Univariate analysis		Multivariate analysis		Univariate analysis		Multivariate analysis	
		HR (95% CI)	*P*	HR (95% CI)	*P*	HR (95% CI)	*P*	HR (95% CI)	*P*
**Age**									
<70	77 (73%)								
≥70	28 (27%)	HR 0.904 (0.455–1.796)	P = 0.773			HR 1.144 (0.565–2.317)	P = 0.708		
**Gender**									
Female	12 (11%)								
Male	93 (89%)	HR 1.239 (0.556–1.866)	P = 0.653			HR 2.734 (0.656–11.39)	P = 0.167		
**Smoking history**									
Never/former	79 (75%)								
Current	26 (25%)	HR 1.096 (0.552–2.176)	P = 0.794			HR 1.315 (0.648–2.668)	P = 0.448		
**Histology** ^a^									
ADC	47 (45%)								
SCC	50 (48%)	HR 0.946 (0.500–1.791)	P = 0.865			HR 1.268 (0.639–2.518)	P = 0.497		
**Histological grade**									
WD	12 (12%)		P = 0.766				P = 0.576		
MD	45 (46%)	HR 0.722 (0.262–1.984)	P = 0.527			HR 0.614 (0.218–1.724)	P = 0.354		
PD	42 (42%)	HR 0.877 (0.324–2.373)	P = 0.796			HR 0.833 (0.305–2.280)	P = 0.723		
**Stage**									
I, II	92 (84%)								
III, IV	13 (16%)	HR 2.066 (0.945–4.520)	P = 0.069	HR 1.877 (0.853–4.134)	P = 0.118	HR 1.759 (0.722–4.285)	P = 0.214	HR 1.638 (0.662–4.053)	P = 0.286
**SIRT1/SIRT2**									
Others	72 (71%)								
Both high	30 (29%)	HR 2.790 (1.150–5.155)	P = 0.001	HR 2.698 (1.457–4.996)	**P = 0.002**	HR 2.280 (1.169–4.449)	**P = 0.016**	HR 2.193 (1.118–4.300)	**P = 0.022**

^a^only the more frequent histological subtypes, adenocarcinomas (ADC) and squamous cell carcinoma (SCC), were analyzed.

WD, well differentiated; MD, moderately differentiated; PD, poorly differentiated. The values highlighted in bold represent significant differences; HR: Hazard ratio.

### SIRT1 and SIRT2 expression affects proliferation of lung cancer cell lines

**Fig 3:**
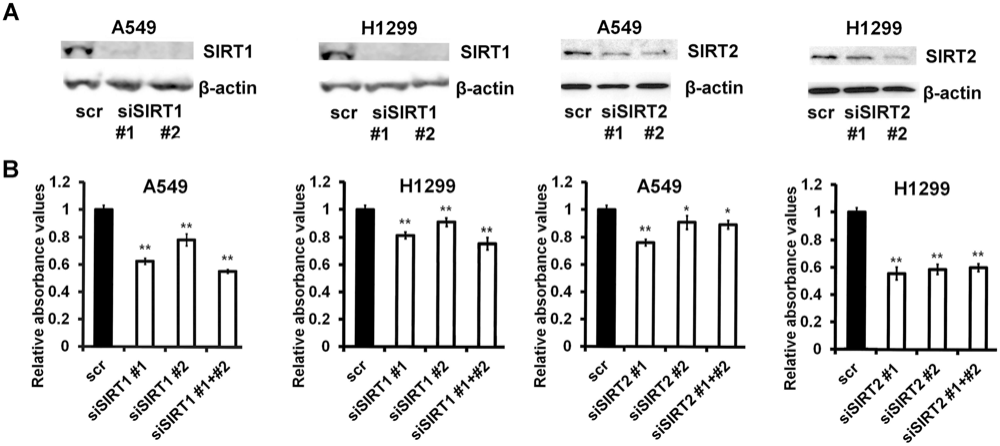
Downregulation of SIRT1 and SIRT2 inhibits proliferation of NSCLC cells. A, immunoblotting of SIRT1, SIRT2 and β-actin in A549 and H1299 cells transfected with scrambled siRNA (scr), siRNA targeting SIRT1 (siSIRT1 #1 or siSIRT1 #2) or SIRT2 (siSIRT2 #1 and siSIRT2 #2). The inhibition was verified after 72 h by Western blotting. B, A549 and H1299 cells were transfected with different siRNAs as indicated. MTT assay was performed 72 h post-transfection. The figure is representative of three different experiments: *, P < 0.05; **, P < 0.01.

In order to investigate the functional role of SIRT1 and SIRT2 in NSCLC, we downregulated their expression in two lung cancer cell lines, A549 and H1299, using two different siRNAs for each gene (Fig 3A). Then we analyzed the effect of SIRT1 and SIRT2 inhibition on cellular proliferation. Anchorage-dependent cell growth, determined by MTT assay, was significantly decreased 72 h post-transfection in both SIRT1- and SIRT2-dowregulated cells in comparison with control cells transfected with a scrambled siRNA (*P*<0.05; Fig 3B).

### Treatment with tenovin-1 reduces proliferation and anchorage independent growth of NSCLC cells

**Fig 4:**
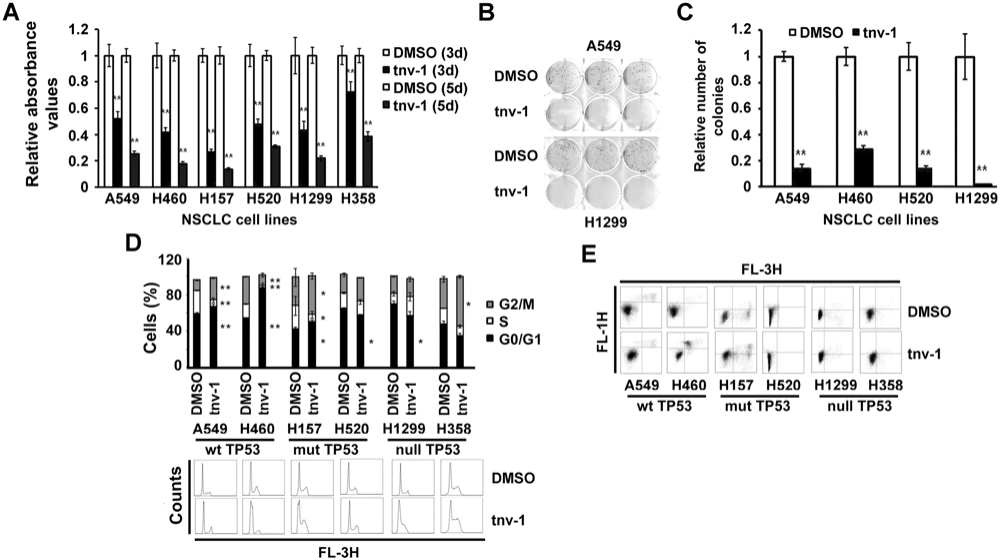
Treatment with tnv-1 decreases growth of NSCLC cell lines. A, NSCLC cell lines were treated with 10 μM tnv-1. After three (3 d) and five (5 d) days the proliferation was measured by MTT assay. Relative proliferation values were obtained by dividing the absorbance values with those of the DMSO controls. Error bars represent standard deviation (SD). **, P < 0.01. B, Representative image of clonogenic assays (anchorage dependent growth). Cells were seeded and treated with tnv-1 (10 μM). After 10 days, colony formation was determined. C, Quantification of soft agar colony formation (anchorage independent growth) after tnv-1 treatment (10 μM) of NSCLC cell lines. Mean relative colony numbers and SD are shown. **, P < 0.01. D, Cell cycle distribution of NSCLC cells. Cells were treated with tnv-1 (10 μM) for 72 h, collected and analyzed by flow cytometry. E, Effect of tnv-1 (10 μM) on apoptosis. Cells were treated (48 h), collected and the cell death was assessed by Annexin V labeling. Error bars, SD.

**Fig 5:**
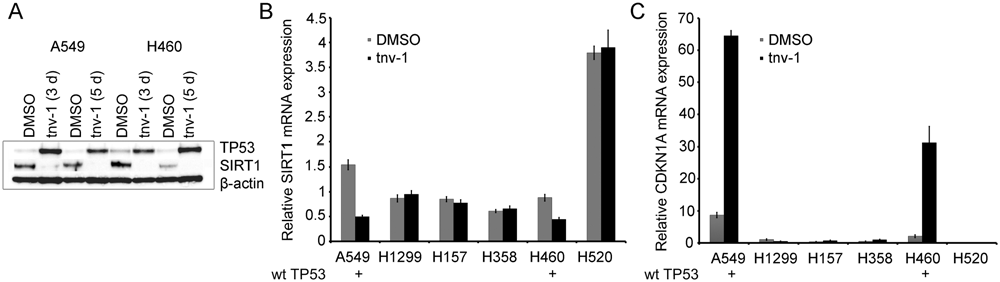
Tnv-1 regulates TP53, SIRT1 and CDKN1A expression levels in TP53 wild-type NSCLC cells. A, TP53 wild-type A549 and H460 cells were treated with 10 μM tnv-1 for three days (3 d) and five days (5 d). Levels of SIRT1 and TP53 were determined by Western blot analysis using β-actin as loading control. B and C, NSCLC cells were treated with 10 μM tnv-1 for 72 hours. Sirtuin 1 (B) and p21 (CDKN1A) mRNA expression (C) was determined by real-time PCR and normalized to IPO8 mRNA levels. Error bars, SD.

We used tnv-1, an inhibitor of SIRT1 and SIRT2 deacetylase activities [19], to analyze the effect of the double inhibition on lung tumor proliferation. We observed that treatment with 10 μM tnv-1 significantly decreased the growth of the six NSCLC cell lines tested (Fig 4A–4C, and S2 Fig). Specifically, at day 5 after the treatment, tnv-1 decreased anchorage-dependent cell growth by ~60–80% (P<0.01; Fig 4A). In the colony formation assay, treatment with tnv-1 (10 μM) also resulted in the inhibition of colony formation in all tested lung cancer cell lines (Fig 4B). Anchorage-independent cell growth in soft agar was also deeply restrained by tnv-1 treatment (P<0.01; Fig 4C). Cell cycle analysis by flow cytometry was also investigated. Treatment with tnv-1 produced a statistically significant increase in the percentage of cells in G1 phase in the TP53 wild-type cell lines A549 and H460 (Fig 4D). In TP53-null NSCLC cell lines (H1299 and H358) and in NSCLC cell lines with mutated TP53 (H157 and H520) we detected diverse effects in the cell cycle phases (Fig 4D and S4 Fig). Moreover, tnv-1 treatment induced cell death in H460 and H157 cells (Fig 4E). In TP53 wild-type A549 and H460 cells, cell cycle arrest in G1 was accompanied by an increase in TP53 and a decrease in SIRT1 levels (Fig 5A). Downregulation of SIRT1 after tnv-1 treatment was further confirmed by real-time PCR (Fig 5B). Moreover, the mRNA levels of *CDKN1A*, a TP53-downstream target, increased in the two cell lines after tnv-1 treatment (Fig 5C). These results are in agreement with previous reports showing transcriptional activation of TP53 after tnv-1 treatment followed by the induction of *CDKN1A* expression and a negative regulation of SIRT1 expression [19,20]. As expected, we observed no effect on SIRT1 or CDKN1A expression after tnv-1 treatment in TP53-mutant cells (Fig 5B and 5C). SIRT2 mRNA expression was not affected by tnv-1, irrespective of the TP53 status (data not shown).

## Discussion

Class III histone deacetylases (HDACs), also called sirtuins, are a group of enzymes involved in numerous cellular processes [21,22]. Despite the growing interest in SIRT1 and SIRT2 research, their function in tumorigenesis is still unclear [2,22]. We have investigated their role in the proliferation of NSCLC cells and their prognostic utility. The data presented here clearly indicate that both sirtuins, SIRT1 and SIRT2, are upregulated in NSCLC at the protein level and that their expression is significantly associated with poor prognosis. Moreover, this is the first study exploring the prognostic value of the simultaneous expression of SIRT1 and SIRT2 in the same cancer patient.

Our results demonstrate that SIRT1 and SIRT2 protein expression is higher in NSCLC cell lines than in non-tumor lung epithelial cells, although these differences were not observed at mRNA level. This fact could be explained to different half-lives of mRNA and protein due to different mechanism including stabilization of SIRT1 mRNA, binding of miRNAs and protein ubiquitylation [23]. Moreover, other mechanisms affecting stabilization by substrate binding may be involved in this process [24]. In this sense, SIRT1 suffers conformational changes upon substrate binding that makes the ternary complex formed by SIRT1, its cofactor NAD+ and the substrate more compact and therefore less accessible to proteolytic degradation [25]. Furthermore, we show here that high levels of SIRT1 are associated with shorter RFS in NSCLC patients. In the recent literature there is an emerging evidence of the role of SIRT1 in carcinogenesis. Increased SIRT1 protein levels have been reported in several malignancies [4,26–30]. In addition, high SIRT1 expression has been proposed as a poor prognostic factor in breast [31,32] and liver carcinoma [33]. In lung cancer patients, in agreement with our results, Tseng *et al*. have demonstrated that those patients with positive SIRT1, low acetylated TP53 and low HIC1 expression have worse prognosis, although the prognostic value of SIRT1 alone was not analyzed in this study [34]. In addition, it has been recently reported that cytoplasmic SIRT1 expression is associated with shorter overall survival in advanced NSCLC [5]. These results are in line with our data, although there is some discrepancy in regards to its subcellular location. SIRT1 was originally identified as a nuclear protein, however some studies have found the protein in the cytoplasm of cancer cells [35]. We have found that the protein was predominantly located in the nucleus although a slight staining could also be observed in the tumor cell cytoplasm of most NSCLC cases.

In the present study we have also shown that SIRT2 protein levels are upregulated in NSCLC cell lines and lung tumor cells, when compared with non-tumor cell lines and tissues, respectively. Accordingly, recent papers support a pro-tumorigenic SIRT2 function in other types of tumors [36,37]. SIRT2 protein levels are significantly higher in hepatocellular carcinoma specimens, relative to normal liver cells, and expression levels correlate with vascular invasion, advanced tumor stages and shorter survival [37]. In agreement with these results, we have found that SIRT2 is a prognostic marker of shorter RFS in NSCLC patients. In contrast to our findings, downregulation of SIRT2 protein has been reported in different malignant tumors [38,39], including lung [10]. The discrepancy could be due to the fact that these studies exploring expression of SIRT2 in lung cancer included non-epithelial (stromal) cells in their scoring algorithm for normal lung, while we have exclusively considered the normal bronchiolar epithelial cells as the normal reference for the comparison with tumor cells.

There are no data in the literature about the prognostic value of the combined evaluation of SIRT1 and SIRT2 protein levels in cancer patients. Our results demonstrate that the combined expression of SIRT1 and SIRT2 is a better predictor of survival than the expression of each one separately.

Our study also revealed that SIRT1 and/or SIRT2 inhibition *via* siRNA hampers cell growth in NSCLC. Our findings are in agreement with previous studies demonstrating that SIRT1 downregulation inhibits proliferation of lung, colorectal, thyroid, breast, prostate and liver cancer cell lines [3,4,26,40–42]. In addition, Liu et al. have recently reported that the repression of SIRT2 decreases cell proliferation in neuroblastoma and pancreatic cells [36].

To confirm the results obtained after siRNA-mediated SIRT1 and 2 silencing, NSCLC cell lines were treated with tnv-1, a SIRT1 and SIRT2 pharmacological inhibitor [19]. We observed that this treatment also inhibited anchorage dependent and independent proliferation of NSCLC cells, confirming that sirtuins play a role in NSCLC cell growth. Also we observed that tnv-1 induced a clear increase in the level of TP53 in TP53 wild-type A549 and H460 cell lines. This increase in TP53 levels was accompanied by a decrease in SIRT1 expression, since SIRT1 transcription can be negatively regulated through two TP53 binding sites present in the SIRT1 promoter [20]. Moreover, tnv-1 treatment also induced CDKN1A (p21) expression and, consequently, G1 phase cell cycle arrest only in TP53 wild-type NSCLC cells. In cell lines without a functional TP53 gene, tnv-1 suppressed cellular proliferation by inducing cell cycle perturbations after the restriction point (H157, H520, H1299 and H358) and apoptosis (H157). It has recently been reported that tenovin-6 (tnv-6) inhibits proliferation of gastric cancer cells in a TP53-independent manner through upregulation of death receptor 5 (DR5) protein [43]. Interestingly, treatment of NSCLC cells H157 (mut TP53) with sirtuin inhibitor salermide also increased DR5 expression and apoptosis [44].

Many mechanisms may be involved in the control of tumor progression by SIRT1/2. Both SIRT1 and SIRT2 are downstream targets of c-MYC oncogene [36,45] and both are part of a positive feedback loop with MYC [36,45]. An attractive hypothesis is that c-MYC could upregulate SIRT1 and SIRT2 protein expression through a post-transcriptional mechanism. Further studies are required to substantiate this speculation. Sirtuins also participate in the epigenetic silencing of several tumor suppressor genes [46]. Moreover, pharmacological inhibition of SIRT1/2 interferes with the Wnt pathway by decreasing the expression of Dishevelled proteins, frizzled 7 receptor (FZD7) and beta-catenin in various cancer cells, which ultimately leads to inhibition of cell growth and cell migration [47,48]. In addition, treatment of TP53-null H1299 cell line with SIRT1/2 inhibitor sirtinol decreases Akt Ser473 phosphorylation, increases Foxo3a levels and triggers apoptosis [49].

Li et al. have shown that tenovins increase apoptosis in chronic myeloid leukemia stem cells and reduce their growth *in vivo* and *in vitro* [50]. Currently, there are active clinical trials investigating the use of inhibitors of class I HDACs (entinostat) or class-I and-II HDACs (vorinostat and panobinostat) in NSCLC therapy. Moreover, there is an active phase I clinical trial with a combination therapy based on pan-deacetylase inhibitors directed against class I, II and III HDACs in lymphoid malignancies (ClinicalTrials.gov Identifier: NCT00691210). Our results support that the class III protein/histone deacetylases inhibition may be a promising strategy for the treatment of NSCLC.

In summary, our results support the hypothesis that SIRT1 and SIRT2 have a protumorigenic role in lung cancer, promoting cell proliferation. Moreover, we have demonstrated herein that the combination of SIRT1 and SIRT2 expression levels is an independent prognostic factor in NSCLC. This observation is reinforced by the finding that blocking SIRT1 and SIRT2 hampers lung cancer cell growth. Together, these results provide a rationale to explore SIRT1 and SIRT2 as prognostic markers and targets for therapy in NSCLC.

## Supporting Information

S1 Fig SIRT1 and SIRT2 mRNA expression in lung cell lines. SIRT1 (A) and SIRT2 (B) mRNA expression was determined by real-time PCR in NSCLC cell lines and HBEC-3KT cells. For endogenous control IPO8 was used. Error bars, SD.
(TIF)

S2 Fig Combination of high levels of SIRT1 and SIRT2 proteins predicts shorter recurrence free survival (RFS) in patients with either adenocarcinoma (ADC) or squamous cell carcinoma (SCC) lung tumors. Kaplan-Meier curves of RFS for patients with high and low immunohistochemical expression of SIRT1 (A, D), SIRT2 (B, E) and the combination of SIRT1 and SIRT2 (C, F), stratifying the whole cohort according to the histological subtype: adenocarcinoma (ADC): A-C; and squamous cell carcinoma (SCC): D-F.
(TIF)

S3 Fig Combination of high levels of SIRT1 and SIRT2 proteins is associated with shorter overall survival (OS) in patients with squamous cell carcinoma (SCC) lung tumors. Kaplan-Meier curves of OS for patients with high and low immunohistochemical expression of SIRT1 (A, D), SIRT2 (B, E) and the combination of SIRT1 and SIRT2 (C, F), stratifying the whole cohort according to the histological subtype: adenocarcinoma (ADC): A-C; and squamous cell carcinoma (SCC): D-F.
(TIF)

S4 Fig Tenovin-1 inhibits cell growth of H358 lung cancer cells. H358 cells were treated with DMSO (control) or tnv-1 (10 μM) and followed by time lapse confocal microscopy. The images were taken using an AxioCam MRm CCD camera (Carl Zeiss) mounted on to a Cell Observer confocal microscope (Carl Zeiss) under 10x magnification (N-Achroplan objective, Carl Zeiss). Photographs were taken every hour (four positions per well) for 72 h. Following acquisition, individual images were processed and converted into time-lapse movies with ImageJ software. The exact cell number per frame was automatically counted by using ImageJ plug-in programmed at CIMA imaging core facility. The relative cell number was obtained by dividing the number of tnv-1 treated cells with the number of control cells at time zero. Lines, relative number of the cells per hour. Error bars, SD. *, P < 0.05; **, P < 0.01.
(TIF)

S1 Table Relationship between SIRT1 or SIRT2 protein expression and clinicopathological features of the NSCLC patients. (DOCX)
