# Supplementary figures and images for: Expression of Sirtuin 1 and 2 Is Associated with Poor Prognosis in Non-Small Cell Lung Cancer Patients
Source: PLoS One. 2015 Apr 27;10(4):e0124670. doi: 10.1371/journal.pone.0124670 (PMC4411155; doi:10.1371/journal.pone.0124670)

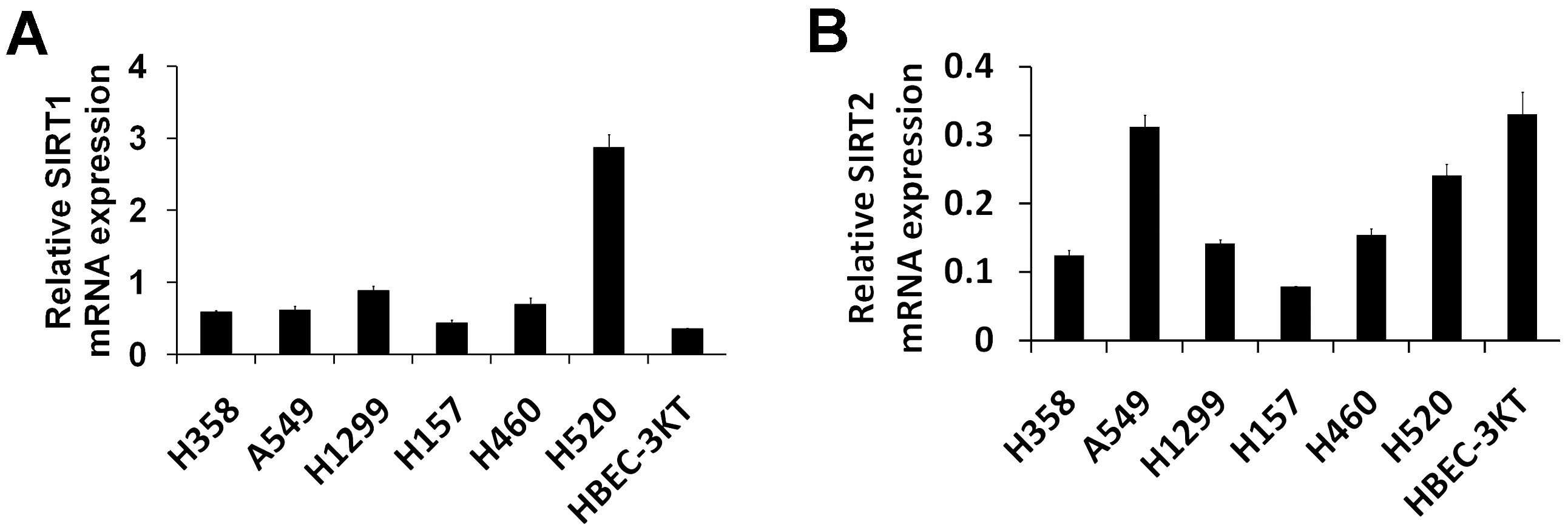

Supplement: S1 Fig — SIRT1 (A) and SIRT2 (B) mRNA expression was determined by real-time PCR in NSCLC cell lines and HBEC-3KT cells. For endogenous control IPO8 was used. Error bars, SD. (TIF) [file pone.0124670.s001.tif]

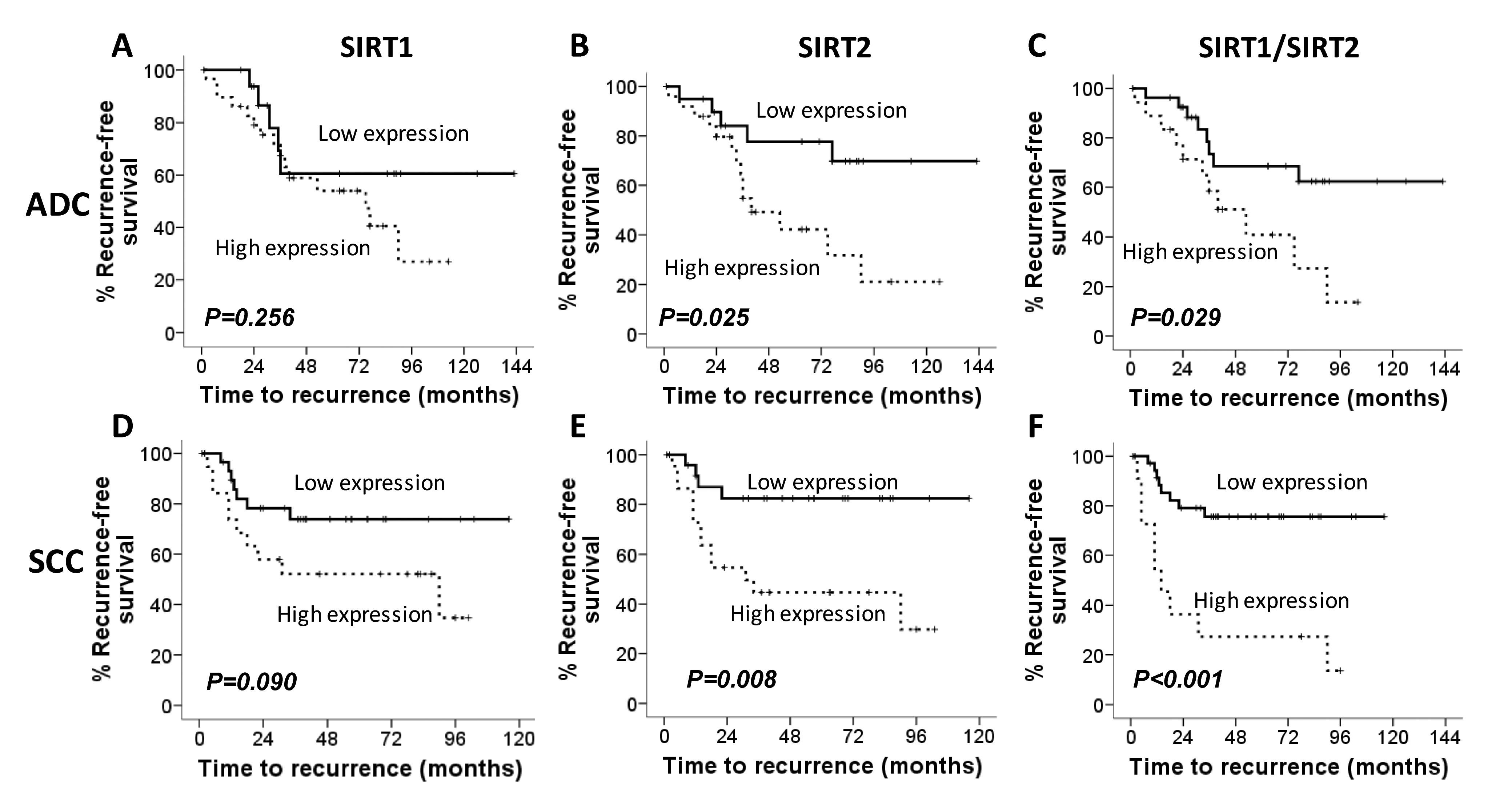

Supplement: S2 Fig — Kaplan-Meier curves of RFS for patients with high and low immunohistochemical expression of SIRT1 (A, D), SIRT2 (B, E) and the combination of SIRT1 and SIRT2 (C, F), stratifying the whole cohort according to the histological subtype: adenocarcinoma (ADC): A-C; and squamous cell carcinoma (SCC): D-F. (TIF) [file pone.0124670.s002.tif]

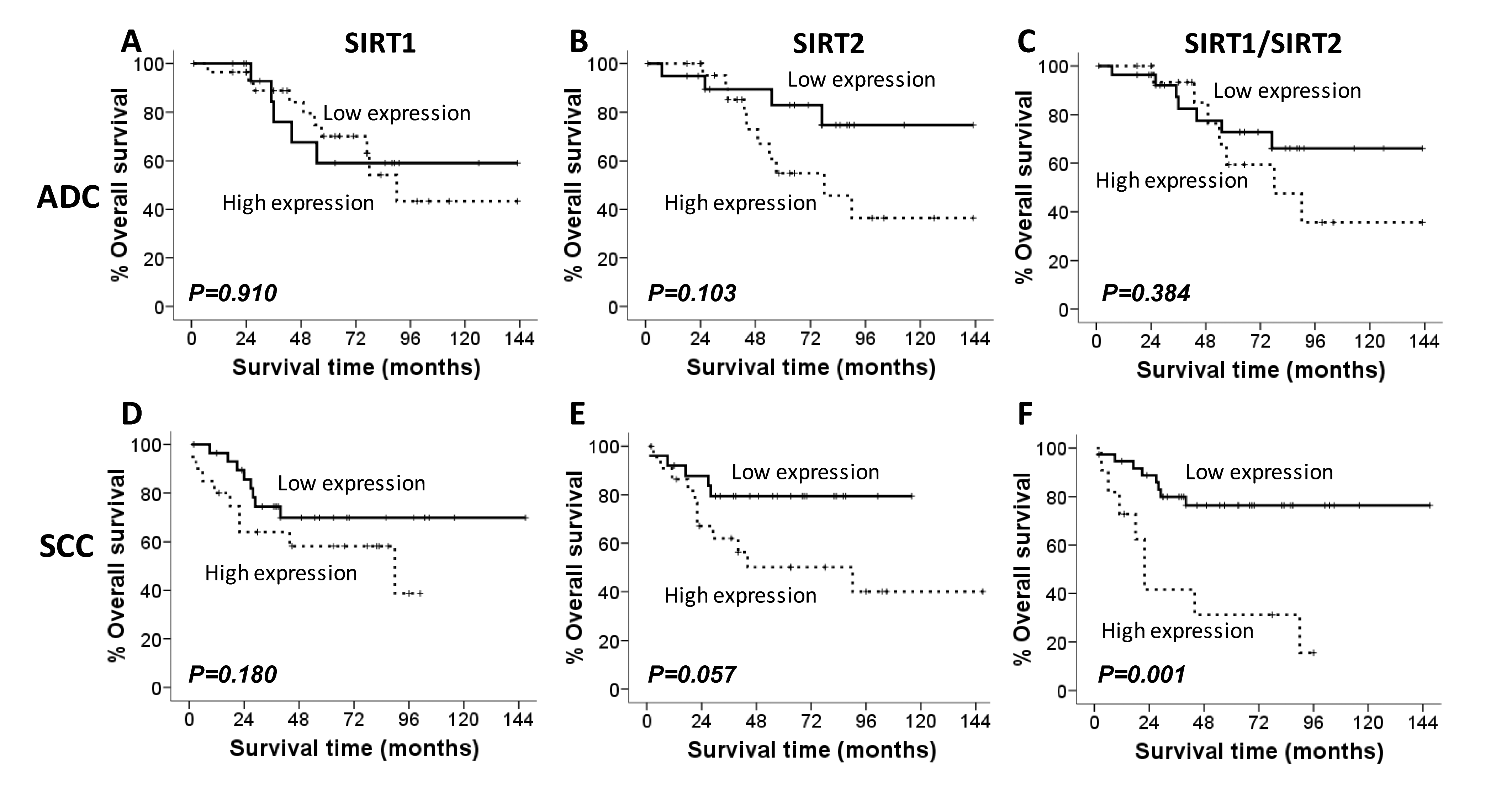

Supplement: S3 Fig — Kaplan-Meier curves of OS for patients with high and low immunohistochemical expression of SIRT1 (A, D), SIRT2 (B, E) and the combination of SIRT1 and SIRT2 (C, F), stratifying the whole cohort according to the histological subtype: adenocarcinoma (ADC): A-C; and squamous cell carcinoma (SCC): D-F. (TIF) [file pone.0124670.s003.tif]

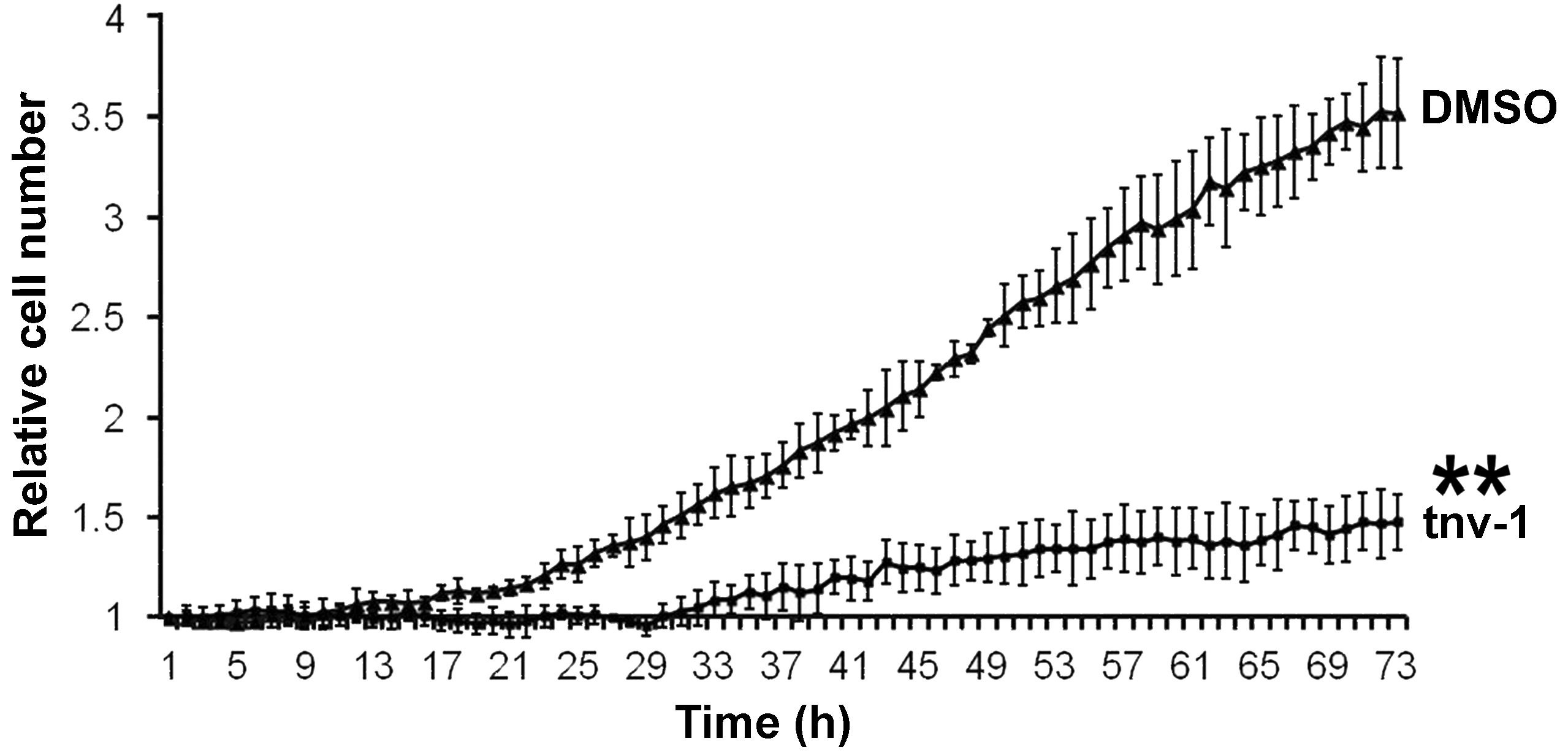

Supplement: S4 Fig — H358 cells were treated with DMSO (control) or tnv-1 (10 μM) and followed by time lapse confocal microscopy. The images were taken using an AxioCam MRm CCD camera (Carl Zeiss) mounted on to a Cell Observer confocal microscope (Carl Zeiss) under 10x magnification (N-Achroplan objective, Carl Zeiss). Photographs were taken every hour (four positions per well) for 72 h. Following acquisition, individual images were processed and converted into time-lapse movies with ImageJ software. The exact cell number per frame was automatically counted by using ImageJ plug-in programmed at CIMA imaging core facility. The relative cell number was obtained by dividing the number of tnv-1 treated cells with the number of control cells at time zero. Lines, relative number of the cells per hour. Error bars, SD. *, P < 0.05; **, P < 0.01. (TIF) [file pone.0124670.s004.tif]
