# Supplementary material for: Expression of Sirtuin 1 and 2 Is Associated with Poor Prognosis in Non-Small Cell Lung Cancer Patients
Source: PLoS One. 2015 Apr 27;10(4):e0124670. doi: 10.1371/journal.pone.0124670 (PMC4411155; doi:10.1371/journal.pone.0124670)
Supplement: S1 Table — (DOCX) [file pone.0124670.s005.docx]

**S1 Table. Relationship between SIRT1 or SIRT2 protein expression and clinicopathological features of the NSCLC patients**

|  | **SIRT1 n (%)** | | | **SIRT2 n (%)** | | |
| --- | --- | --- | --- | --- | --- | --- |
|  | **Low** | **High** | ***P*** | **Low** | **High** | ***P*** |
| **Age (years)** |  |  |  |  |  |  |
| <70 | 38 (49.4) | 39 (50.6) |  | 36 (48) | 39 (52) |  |
| ≥70 | 15 (53.6) | 13 (46.4) | 0.702 | 13 (48.1) | 14(51.9) | 0.989 |
| **Gender** |  |  |  |  |  |  |
| Male | 49 (52.7) | 44 (47.3) |  | 46 (50.5) | 45(49.5) |  |
| Female | 4 (33.3) | 8 (66.7) | 0.207 | 3 (27.3) | 8 (72.7) | 0.144 |
| **Histology^a^** |  |  |  |  |  |  |
| ADC | 18 (38.3) | 29 (61.7) |  | 20 (43.5) | 26 (56.5) |  |
| SCC | 30 (60) | 20 (40) | **0.033** | 25 (52.1) | 23 (47.9) | 0.404 |
| **Stage** |  |  |  |  |  |  |
| I-II | 48 (52.2) | 44 (47.8) |  | 42 (47.2) | 47 (52.8) |  |
| III-IV | 5 (38.5) | 8 (61.5) | 0.355 | 7 (53.8) | 6 (46.2) | 0.654 |
| **Nodal (N) stage** |  |  |  |  |  |  |
| N0 | 41 (54.7) | 34 (45.3) |  | 36 (50) | 36 (50) |  |
| N1-N2 | 12(40) | 18 (60) | 0.392 | 13 (43.3) | 17 (56,7) | 0.539 |
| **Histological grade** |  |  |  |  |  |  |
| WD | 8 (66.6) | 4 (33.4) |  | 4 (36.4) | 7 (63.6) |  |
| MD | 25 (55.5) | 20 (44.5) |  | 20 (46.5) | 23 (53.5) |  |
| PD | 19 (45.2) | 23 (54.8) | 0.364 | 21 (50) | 21 (50) | 0.721 |
| **Smoking history** |  |  |  |  |  |  |
| Never/Former | 40 (50.6) | 39 (49.4) |  | 39 (50.) | 39 (50) |  |
| Current | 13 (50) | 13 (50) | 0.955 | 10 (41.7) | 14 (58.3) | 0.475 |

WD, well differentiated;MD, moderately differentiated; PD, poorly differentiated.

^a^ only the more frequent histological subtypes, adenocarcinomas (ADC) and squamous cell carcinoma (SCC), were analyzed.

|  |  | |  |  | | |  | |
| --- | --- | --- | --- | --- | --- | --- | --- | --- |
|  |  |  |  |  |  | |  |  |
|  |  |  |  |  | |  | |  |
|  |  |  |  |  | |  | |  |
|  |  |  |  |  | |  | |  |
|  |  |  |  |  | |  | |  |
|  |  |  |  |  | |  | |  |
|  |  |  |  |  | |  | |  |
|  |  |  |  |  | |  | |  |
|  |  |  |  |  | |  | |  |
|  |  |  |  |  | |  | |  |
|  |  |  |  |  | |  | |  |
|  |  |  |  |  | |  | |  |
|  |  |  |  |  | |  | |  |
|  |  |  |  |  | |  | |  |
|  |  |  |  |  | |  | |  |
|  |  |  |  |  | |  | |  |
|  |  |  |  |  | |  | |  |
|  |  |  |  |  | |  | |  |
|  |  |  |  |  | |  | |  |
|  |  |  |  |  | |  | |  |
|  |  |  |  |  | |  | |  |
|  |  |  |  |  | |  | |  |
|  |  |  |  |  | |  | |  |
|  |  |  |  |  | |  | |  |
|  |  |  |  |  |  | |  | |
